# Primary intraosseous meningioma: atypical presentation of a common tumor

**DOI:** 10.1590/0100-3984.2017.0106

**Published:** 2018

**Authors:** Benardo Carvalho Muniz, Bruno Niemeyer de Freitas Ribeiro, Nina Ventura, Emerson Leandro Gasparetto, Edson Marchiori

**Affiliations:** 1 Instituto Estadual do Cérebro Paulo Niemeyer - Departamento de Radiologia, Rio de Janeiro, RJ, Brazil.; 2 Universidade Federal do Rio de Janeiro (UFRJ), Rio de Janeiro, RJ, Brazil.

Dear Editor,

A 41-year-old woman presented with an approximately one-year history of progressive facial swelling and left-sided visual impairment. A computed tomography (CT) scan of the skull showed a sclerotic, expansile lesion on the lateral/upper wall of the left orbit, narrowing and extending to the optic canal. Magnetic resonance imaging (MRI) showed a lesion with a hypointense signal in T1-weighted and T2-weighted sequences, without significant contrast uptake, accompanied by a slight contrast-enhanced thickening of the subjacent dura mater, which was compressing the left optic nerve. A histopathological study confirmed the suspected diagnosis of intraosseous meningioma.

Recent studies in the radiology literature of Brazil have emphasized the importance of imaging examinations in improving the diagnosis of central nervous system disorders^(^^1^^-^^3^^)^. Meningioma is the most common primary intracranial tumor, representing approximately 14-20% of cases. The vast majority are intradural lesions, extradural lesions accounting for only 1-2%^(^^4^^)^. Extradural meningiomas affect the cranial vault in 68% of cases, such lesions being referred to as primary intraosseous meningiomas (PIMs), which mainly affect the frontal and parietal bones, as well as the region of the orbit^(^^5^^-^^7^^)^. Other common locations for extradural involvement are the subcutaneous tissue, paranasal sinuses, and parapharyngeal spaces, as well as, in rare cases, the lungs and adrenal glands^(^^5^^,^^6^^)^. Unlike typical intradural meningiomas, which primarily affect females between the ages of 50 and 69 years and usually have a benign course, PIMs can affect either gender, have a peak incidence in the second decade of life, and are more likely to evolve to malignant degeneration^(^^6^^)^.

**Figure 1:**
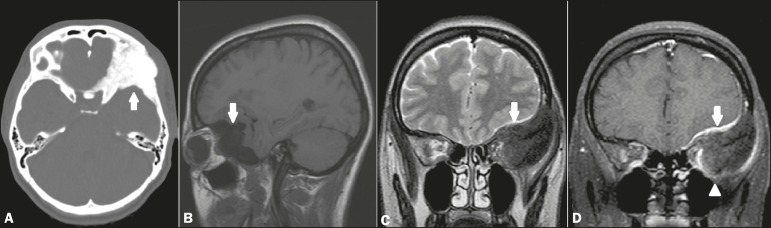
**A:** Axial CT scan, with bone window settings, showing an expansile, osteoblastic lesion, affecting the upper/lateral wall of the left orbit (arrow). **B:** Noncontrast sagittal T1-weighted MRI sequence, showing a lesion with a hypointense signal (arrow). **C:** Coronal T2-weighted MRI sequence, also showing the lesion with a hypointense signal (arrow). Also note the compressive effect and displacement of the intraorbital structures, including the optic nerve. **D:** Contrastenhanced coronal T1-weighted MRI sequence, showing a lack of significant contrast uptake within the lesion (arrowhead), with only slight uptake in the dura mater subjacent to the tumor (arrow).

On CT, most PIMs (65%) present as expansile, osteoblastic bone lesions, with or without cortical destruction^(^^6^^)^. On MRI, they commonly hypointense in T1- and T2-weighted sequences, typically without significant contrast enhancement, as in the case reported here^(^^5^^)^. However, in rarer cases, if a PIM presents as an osteolytic lesion on CT, an MRI scan can show a hypointense signal in T1-weighted sequences and a hyperintense signal in T2-weighted sequences, as well as contrast enhancement^(^^6^^,^^7^^)^. Although PIMs do not present the dural tail sign that is often found in intradural meningiomas, there can be contrast uptake in the dura mater subjacent to the tumor due to venous stasis or to tumor invasion, as demonstrated in our case^(^^7^^)^. There are inherent differences between CT and MRI, the former allowing better delineation of bone involvement, whereas the latter provides a better assessment of the soft-tissue involvement and extradural extent of the lesion^(^^6^^)^.

The differential diagnosis of osteoblastic PIM includes typical intradural meningioma with reactive hyperostosis, in which the meningeal component of the lesion is the most obvious. Other diagnoses that should be considered are metastases, plasmacytoma, fibrous dysplasia, osteoma, osteosarcoma, and Paget’s disease^(^^6^^)^.

In most cases of PIM, the treatment is total surgical resection, with subsequent cranial reconstruction. If the resection is partial, there should be radiological follow-up; if the disease has recurred or if the residual lesion has progressed, the next surgical procedure can be accompanied by adjuvant radiotherapy^(^^6^^)^.

In conclusion, although rare, PIMs should be considered in the differential diagnosis of bone lesions, especially when the lesions are osteoblastic and located in the cranial vault.
